# Insight inTo Stress and POOping on Work TIME (ITS POO TIME): Protocol for a Web-Based, Cross-Sectional Study

**DOI:** 10.2196/58655

**Published:** 2025-06-05

**Authors:** Phillip John Tully, Suzanne Cosh, Gary Wittert, Sean Martin, Andrew Vincent, Antonina Mikocka-Walus, Deborah Turnbull

**Affiliations:** 1 Faculty of Health and Medical Sciences Freemasons Centre for Male Health and Wellbeing The University of Adelaide Adelaide Australia; 2 School of Psychology Deakin University Waurn Ponds Australia; 3 School of Psychology The University of Adelaide Adelaide Australia; 4 Australian Institute of Family Studies Southbank Australia; 5 Deakin University School of Psychology Burwood Australia

**Keywords:** disorders of gut-brain interaction, gastrointestinal disease, irritable bowel syndrome, shift work, anxiety, occupational health, occupational stress, parcopresis, social anxiety disorder, mixed-methods

## Abstract

**Background:**

Long occupational working hours and shift work are common in high-, middle-, and lower-income economies. Bowel movement frequency and stool form in occupational settings may be important markers of stressful working conditions as well as diurnal gut microbiota action, gastrointestinal discomfort, and disorders of gut-brain interaction (DGBI). Characterizing DGBI in shift and nonshift workers could help identify the impact of diurnal work patterns on workers’ physical and mental health, including anxiety related to bowel movements.

**Objective:**

This study aims to outline the Insight inTo Stress and POOping on work TIME (ITS POO TIME) protocol describing a web-based multimethods research project on DGBI, stool form and frequency, psychological factors, sleep, diet, and anxiety related to bowel movements in occupational settings by comparison to residential settings.

**Methods:**

Study 1 comprises a web-based convenience sampling survey to acquire quantitative data from adults who are engaged in paid employment. We seek to assess occupational characteristics, organizational factors, as well as standardized questionnaires for stool form, DGBI assessed based on Rome-IV criteria, sleep, diet, bowel movement anxiety (ie, parcopresis), and distress. Study 2 is a qualitative study that asks open-ended questions about respondents’ attitudes to defecation at work. Analyses for study 1 will explore rates of DGBI in shift versus nonshift workers and explore how occupational characteristics are associated with occupational bowel movement stool form and frequency. With regards to distress and parcopresis, study 1 will analyze how parcopresis, distress, and contamination fears are associated with stool form and frequency in occupational settings compared with residential settings. Study 1 is designed to have 90% power to detect a 5% difference in DGBI prevalence between groups at α=.05 based on the conservative estimate of 15% DGBI prevalence in shift workers and 10% DGBI prevalence in nonshift workers, with a final sample of 1967 required. Study 2 qualitative data will be analyzed using inductive thematic analysis to identify themes concerning feelings and attitudes about bowel movements in occupational settings.

**Results:**

The findings of ITS POO TIME will elicit important information on what factors are associated with bowel movements and stool form and frequency in occupational settings and identify associations pertinent to occupational health. Data collection commenced in January 2019 and finished enrollment in December 2023. Study 1 obtained 1872 responses and fell short of the desired sample size. Study 2 received 337 responses, and the primary results are expected to be published in 2025 and the qualitative results published in 2026.

**Conclusions:**

The results of the research described in this research protocol will have direct implications for industry, employers, and policy makers concerning DGBI, stress, and worker health.

## Introduction

Extended occupational hours including paid and unpaid overtime as well as shift work during the biological night are an increasingly vital and common component of the labor force in global economies. The number of shift workers alone is estimated at 22 million in the United States [1]. Long work hours and shift work may lead to disrupted circadian rhythms [2], change in hormonal metabolism [3], change in diet [4,5], and irregular timing of eating [6] as a direct consequence of being exposed to artificial light at night [7,8]. The influence of diurnal rhythms on gut microbiota [9,10], patterns of food intake, and satiety [11], is being increasingly recognized as important to worker health. Direct health consequences include upregulated inflammatory response and metabolic disease [10,12], obesity [13], gastrointestinal diseases, and disorders of gut-brain interaction (DGBI) [14]. This research predominantly relates to the latter aspect of gastrointestinal health, specifically, DGBI such as irritable bowel syndrome and related symptoms as they are among the most common health complaints of shift workers [15]. Other DGBI can be categorized as esophageal (eg, functional heartburn), gastroduodenal (eg, functional dyspepsia), bowel (eg, functional diarrhea), centrally mediated disorders of gastrointestinal pain, gallbladder and sphincter of Oddi disorders (eg, functional gallbladder disorder), anorectal disorders (eg, fecal incontinence), as well as childhood functional gastrointestinal disorders.

A higher prevalence of gastrointestinal diseases, DGBI, and related symptoms documented among shift workers than among their nonshift worker peers include *Helicobacter pylori* infection [16,17], duodenal ulcer [18], irritable bowel syndrome [19-21], constipation [22,23], diarrhea, abdominal bloating, gastroesophageal reflux, and regurgitation [23]. A high prevalence of gastrointestinal disease and DGBI is reported in diverse shift work samples such as Iranian petrochemical workers [24], US air traffic controllers [25], German resident physicians [26], Indian restaurant workers [27], Danish fishermen [28], and merchant sailors [29]. High gastrointestinal disease and DGBI rates among nurse shift workers alone were reported in Brazil [30], South Korea [21], Iran [23], Iceland [31], Poland [22], and Turkey [32]. Collective findings raise the likelihood that shift work, regardless of occupation or location, is associated with DGBI. However, most research to date is merely descriptive of gastrointestinal symptom correlates in different occupational settings, and despite this, our understanding of putative associations remains incomplete.

One aspect of gastrointestinal disease and DGBI risk in shift workers that has not been examined comprehensively is the association between gastrointestinal symptoms and defecation routines in occupational settings. Changes in defecation patterns during shift work could be an important marker of diurnal gut microbiota action, gastrointestinal discomfort, or an early indicator for DGBI [33]. Indeed, shift workers may face high job demands as well as limited access to toilets and toilet breaks in addition to the abovementioned diurnal changes [34]. A parallel body of research in school settings offers insights into the impact of restricting access to toilets and voiding behaviors [35-37]. A survey of 791 school teachers suggested that teachers restricted their fluid intake to limit the number of toilet breaks and this was associated with a higher likelihood of experiencing urinary tract infections [37]. Similarly, school students reported behavioral strategies including limiting food and water intake to mitigate the need to use school toilets [38]. Students have also reported avoiding bowel movements at school due to a lack of privacy and perceptions of uncleanliness [38-40]. The ratio of toilets to users has also been identified as a factor in toilet use among students and workers, with privacy and uncleanliness again cited as reasons for toilet avoidance behaviors [35,39]. Despite these findings, there is a very limited understanding of whether such avoidances persist into adulthood and whether toilet access impacts worker attitudes and defecation behaviors in occupational settings. To the best of our knowledge, no study has investigated symptoms of DGBI, occupational toilet characteristics, and attitudes to defecation in occupational settings in shift and nonshift workers.

A second aspect of the association between shift work and gastrointestinal health that remains unclear is the role of psychosocial factors such as stress. Occupational health policies typically focus on biological, chemical, physical, and ergonomic factors, paying less attention to the psychosocial well-being of their workers [12,41,42]. To date, research on the interplay between shift work and gastrointestinal symptoms has largely neglected psychosocial factors. This approach belies prior reports linking shift work with depression [43-45], suicidal ideation [45], burnout [46], workplace conflict [30], poorer decision-making [47,48], workplace accidents [12], and disruption to life outside work [49]. Similarly, psychosocial factors like stress are implicated in DGBI such as irritable bowel syndrome [50-56]. The collective findings raise the possibility that stress is a contributing factor in the association between shift work and adverse gastrointestinal symptoms such as those characterizing DGBI.

Indeed, the term stress is rather nebulous, and therefore it is imperative to further explore different stressors that may include general distress, anxiety, and even voiding-specific stressors. Specifically, diagnostic descriptors of social anxiety disorder refer to “bladder shyness” and difficulties using public bathrooms [57]. More specifically, parcopresis is a term used to describe anxiety surrounding one’s own bowel movements in public restrooms because of an overwhelming fear of perceived scrutiny [58]. Given that prior research indicates approximately 85% of students avoid defecating at high school [59], it remains unknown whether such avoidance behaviors and social concerns persist into adulthood and occupational settings. Elucidating defecation avoidance behaviors in adult workers is imperative because such avoidances may have harmful secondary consequences to gastrointestinal health. Unfortunately, less empirical data exist concerning parcopresis by comparison to social anxiety disorder in general as well as paruresis, that is, the difficulty or inability to initiate or sustain micturition [60]. Conceptual models of social anxiety applied to parcopresis and paruresis [61] support the direct contribution to symptom severity from the components of the extended bivalent fear of evaluation model [62]; fear of negative and positive evaluation, concerns of social reprisal, and dysfunctional attitudes [63]. Parcopresis was also associated with toilet avoidance more commonly than contamination fears (17% vs 3%) underscoring that social anxiety processes were responsible for toilet avoidance behaviors [64]. Further empirical data on parcopresis could help improve understanding of this condition contextualized against socio-occupational toileting norms. Specifically, it is unclear if parcopresis symptoms are heightened in restrooms used by work colleagues where perceived social scrutiny could foreseeably be higher. Most research to date investigates toilet behaviors in public restrooms that are largely used by strangers and not work colleagues. To the best of our knowledge, neither social anxiety nor parcopresis has been examined in relation to bowel habits in occupational settings.

The Insight inTo Stress and POOping on Work TIME (ITS POO TIME) study’s main aim is to comprehensively describe the prevalence of probable DGBI in shift- and nonshift workers, estimate the prevalence of parcopresis in adult workers, and examine the impact and characteristics of residential and occupational bowel movements. A secondary aim is to explore the role of pertinent covariates including sleep, diet, and psychosocial stress while also collecting data on relevant occupational characteristics. A qualitative study aims to identify attitudes to bowel movements in occupational settings.

## Methods

### Study 1

#### Recruitment

The quantitative study uses a cross-sectional survey and convenience sampling design, hosted on the SurveyMonkey electronic platform (Symphony Technology Group). Participants are recruited via advertising at The University of Adelaide Medical School, The University of New England (UNE) Faculty of Health, as well as investigator promotional interviews with national radio and free-to-air television stations, and organizations representing occupations with shift work (Nursing Times) or known toileting restrictions (NSW Teachers Federation). The study adheres to the principles of the Checklist for Reporting Results of Internet E-Surveys (CHERRIES) [65] and is reported in Multimedia Appendix 1.

#### Ethical Considerations

This study was approved by the Human Research Ethics Committees of the University of Adelaide (H-2018-235) and UNE (HE18-300). Study participants provided informed consent and could opt out of the study at any time by closing their browser window. All submitted data was anonymized and deidentified and respondents did not receive any compensation for their participation.

Respondents reporting any concerns to the questions pertaining to gastrointestinal health are provided with referral information for national health care services and recommendations to consult their primary care provider in the first instance. Participants reporting any concerns relating to mental health are provided with 24-hour emergency and general mental health support services.

#### Statistical Analysis

Study 1 is designed to have 90% power to detect a 5% difference in DGBI prevalence between groups at α=.05 (1-sided) based on the conservative estimate of 15% DGBI prevalence in shift workers and 10% DGBI prevalence in nonshift workers [15]. The study aimed to recruit 2500 persons based on the anticipated sampling of 25% shift workers and 75% nonshift workers, allowing for 25% incomplete responses in the study, with a final sample of 1967 required.

Hypothesis 1: There will be higher levels in occupational stool form indicating looser stools when compared with residential stools.

Hypothesis 2: Shift workers will have increased odds for DGBI compared with persons not engaged in shift work.

Hypothesis 3: Higher anxiety and distress scores will be observed in persons with DGBI.

Hypothesis 4: Higher parcopresis anxiety symptoms will be more strongly associated with bowel movement form in occupational settings than occupational characteristics.

To assess hypothesis 1, a paired-sample test will be used to evaluate the change between residential and occupational stool types rated on the Bristol Stool Chart. To assess hypothesis 2, analyses will use logistic regression showing the odds ratios and 95% CIs for any probable DGBI (binary outcome) in shift workers in comparison with their nonshift working counterparts using nonadjusted, minimally adjusted (sociodemographic factors), and adjusted for occupational factors such as perceived toilet break adequacy and other self-reported measures including distress. To assess hypothesis 3, a 1-way ANOVA will examine if higher anxiety and distress scores are observed in persons with DGBI compared with their non-DGBI counterparts, using nonadjusted, minimally adjusted models (sociodemographic factors), and models fully adjusted for occupational factors. To assess hypothesis 4, multivariable regression will assess the association between occupational bowel movement type (outcome) and parcopresis (predictor) and occupational factors (predictors), adjusted for other self-reported measures including contamination fears. Occupational factors include toilet break adequacy, number of toilets, and toilet subtype, perceived adequacy of toilet breaks.

#### Eligibility

Inclusion criteria were individuals aged ≥18 years who self-identify as having paid employment in a part-time or full-time capacity in any work role in any organization, or self-employed, or undertaking paid training (eg, apprentice and internist).

Exclusion criteria were individuals who were retired, had a volunteer role, had unpaid work placement, were working from home office (eg, during COVID-19 lockdowns), were working in a sheltered workshop due to intellectual disability or developmental disorder, had no access to the internet to complete the survey, and were unable to visually rate one’s own stool.

#### Measures

##### Demographic and Occupational Data Collected

General descriptive information included age, gender, occupation, industry, years of employment in current job, household income, shift work type, the number of employees at each workplace, number of toilets at the place of employment, type of toilet (gender-specific, gender-neutral, or shared), cubicle within larger toilet room or enclosed blocks, and perceived adequacy of toilet breaks. We will also quantify how often respondents report defecating in occupational and residential settings.

##### Medical History

Self-report questions (dichotomized, yes or no) ask about the past history of gastrointestinal surgery, gastrointestinal cancer, urological surgery, urological cancer, spine surgery, use of laxatives, and use of antidiarrhea drugs. This serves as the prescreener before administering comprehensive questions pertaining to gastrointestinal health and DGBI.

##### Shift Work

We will use reference values for melatonin and the biological night (9 PM-7 AM) as proposed by Arendt and the UK Labour Force Survey [66] to classify workers using their self-reported typical start and end work times as shift work or nonshift work.

##### Gastrointestinal Disorders

The primary outcome of interest is the prevalence of probable DGBI measured by the Rome IV Diagnostic Questionnaire (R4DQ) [67]. The R4DQ was designed to measure esophageal disorders, gastroduodenal disorders, bowel disorders, centrally mediated disorders of gastrointestinal pain, gall bladder and sphincter of Oddi disorders, and anorectal disorders, as well as two domains not relevant to the proposed project (excluded: childhood functional disorders in neonates or toddlers or children or adolescents). The questionnaire asks respondents a series of questions about gastrointestinal symptoms over the past 6 months, and between 26 to 86 questions are asked in total depending on R4DQ skip pattern logic that we implemented for ITS POO TIME. It takes respondents approximately 15 minutes to complete the R4DQ. Validation studies with the R4DQ [67] indicate high receiver operating characteristic specificity for the detection of any irritable bowel syndrome, functional dyspepsia, and functional constipation (specificity 97.1%, 93.3%, and 93.6%, respectively). However, the sensitivity for detection of DGBI’s was low irrespective of whether disorders were mutually exclusive (<63%) or when disorders were permitted to overlap (73%) [67]. In Australia, the R4DQ was successfully administered via the web to 2036 Australians as part of a study on the global prevalence of DGBI [68].

##### Stool Form and Frequency

Using the Bristol Stool Chart [69], participants were asked to identify the form and frequency of their most recent stool. There are 7 options on the Bristol Stool Chart, with 2 categories indicative of constipation, two categories indicative of normal bowel movements, and 3 categories indicative of diarrhea or urgency. The Bristol Stool Chart was adapted here to ask respondents to rate their most recent stool in the occupational setting, where we obtained information about toilet design. A modified Bristol Stool Chart was also asked for respondents to rate their most recent residential stool. Other bowel habit questions were adapted from the Taiwan Teacher Bladder Survey that asked participants about the adequacy of toilet breaks and frequency of toilet behaviors [36].

##### Sleep

The 8-item Sleep Condition Indicator (SCI) asks respondents about their concerns about getting to sleep, remaining asleep, sleep quality, daytime personal functioning, daytime performance, duration of sleep problem, nights per week having a sleep problem, and extent troubled by poor sleep. The SCI has high internal consistency (α≥.86) and convergent validity with other sleep measures [70].

##### Diet

General food frequency was quantified by an abbreviated Food Frequency Questionnaire (FFQ). Abbreviated FFQs are common in epidemiological surveys when diet is not the key variable of interest. We selected 10 items from the 2008/2009 New Zealand nutrition survey that asks about the frequency of eating fruit (servings per day), vegetables (servings per day), wholemeal or whole grain foods, soft drinks, alcohol (standard units), cake sweet or chocolate biscuits, processed meats, red meat, low or reduced fat foods, take away foods [71].

##### Parcopresis

Several scales exist for anxiety related to urinary voiding; however, only one exists for anxiety related to bowel movements. Toilet anxiety in relation to bowel movements was quantified by the 8-item parcopresis subscale of the Shy Bladder and Bowel Scale [72]. The questionnaire was explicitly designed to assess toilet anxiety in adults and consists of 2 subscales, one for urinary voiding and one for bowel voiding. A validation study of the Shy Bladder and Bowel Scale was performed in psychology students (n=387) and general population groups (n=334), which supported the proposed 8-item 2-factor structure as well as favorable content, concurrent, and test-retest validity [72]. The scale asks respondents a series of questions about their toilet behaviors on a Likert-type scale from “none of the time,” “a little of the time,” “some of the time,” “most of the time,” and “all of the time.” Higher scores are indicative of higher parcopresis.

##### Social Anxiety

Social anxiety was quantified with 3 items from the abbreviated Social Phobia Inventory (Mini-SPIN) [73]. The Mini-SPIN items are “Fear of embarrassment causes me to avoid doing things or speaking to people,” “I avoid activities in which I am the centre of attention,” and “Being embarrassed or looking stupid are among my worst fears.” Respondents rate each item on a Likert-type scale from “not at all,” “a little bit,” “somewhat,” “very much,” and “extremely.” The Mini-SPIN was evaluated in 344 persons in a 2-stage psychiatric assessment, which showed that a cutoff score of ≥6 demonstrated high sensitivity (88.7%), specificity (90%), and negative predictive value (98.5%), supporting its utility as a screening tool for social anxiety disorder.

##### Contamination Fears

Fear of contamination was quantified with 3 items comprising the contamination subscale of the Obsessive-Compulsive Inventory-Revised as validated by Huppert et al [74]. The subscale items are “I find it difficult to touch an object when I know it has been touched by strangers or certain people,” “I sometimes have to wash or clean myself simply because I feel contaminated,” and “I wash my hands more often and longer than necessary.” Respondents rate each item on a Likert-type scale from; “not at all,” “a little,” “moderately,” “a lot,” and “extremely.”

##### Distress

General distress is measured with the Kessler Psychological Distress Scale (K10) [75]. The 10-item questionnaire asks about general psychological distress in the previous month. Respondents rate each of the 10 symptoms on a Likert scale from 1 “none of the time” to 5 “all of the time.” The K10 is commonly used in epidemiological surveys and normative data exist for the Australian general population [76]. Scores range from 10 to 50, with cutoff scores of 10 to 15, 16 to 29, and ≥30 indicative of low, medium, and high risk for a probable mood or anxiety disorder.

### Study 2

#### Recruitment

Study 2 is a distinct study hosted on Qualtrics and was advertised concurrently with study 1, and it was possible to participate in both or just study 1 or study 2. Advertising was performed through the UNE Faculty of Health research participant pool. UNE typically attracts students characterized as older adult learners retraining for a new career who are engaged in part- or full-time work in addition to their university studies.

#### Ethical Considerations

Study 2 was approved by the Human Research Ethics Committee of UNE (HE19–198). The study is anonymous, and participants can withdraw at any time. Any potentially identifying information (eg, name of the workplace) will be removed from any publication of results or data sharing.

#### Methods and Analysis

The study will be conducted in line with the well-recognized quality principles for qualitative research outlined by Tracy [77], including rigor and sincerity. For example, we will aim to provide sufficient, abundant, appropriate, and complex data to ensure rigor. To achieve this, data analysis will be iterative and conducted by multiple authors. Doing so enables peer discussion and reflection in order to engage with reflexive considerations and manage researcher subjectivity and theoretical assumptions [78]. Ongoing engagement with the data through the analysis further enables reflexivity to be explored and considered. To achieve sincerity, we will provide a reflexivity statement about the relevant background details of our team so that the reader can judge how these may have impacted on our approach to the work, including the way that we have interpreted the data. Ongoing review of analysis and the coded data by multiple authors will also take place to support analytic rigor and trustworthiness by ensuring that analysis provides a clear and coherent representation of the data, with trustworthiness and openness also supported by the presentation of detailed extracts in publications from the study.

Qualitative data will be analyzed using an inductive thematic analysis at the latent level from a realist epistemology perspective [79]. Thematic analysis can be viable on open-ended survey questions, and data will be coded across the dataset, rather than by individual questions, to ensure breadth and depth of patterns across the entirety of the data [76]. Study analysis will expand on study 1 results regarding workplace parcopresis by further outlining attitudes and experiences of defecation in the workplace. Data will be read and reread to gain familiarity. Initial coding of data will be undertaken, with similar codes grouped together. Coding will be conducted across the entire dataset rather than individual items, as recommended for qualitative online survey research [80]. In the next stage of analysis, data for each code will be collated and reviewed in depth, with a view to exploring patterns and themes in the data. Preliminary themes and the generated codes will be reviewed by other authors. In an iterative process, overarching themes and subthemes will be reviewed and refined to ensure coherence and distinctiveness, so that they provide a comprehensive representation of the data.

The quantitative (study 1) and qualitative data (study 2) will be analyzed separately. Due to the deidentified nature of the study, it is not possible to determine if a respondent participated in both studies or not. Thus, an integrated analysis of the 2 datasets (eg, outliers and typical instances from the quantitative findings) cannot be performed. Study 2 will build on the results of study 1 by providing in-depth insight into experiences around defecation at work to complement quantitative findings.

#### Power

While it is not possible to ascertain the power of qualitative research, and some coherently argue against such a quantitative approach to inductive thematic analysis [81], nonetheless, a number of factors can guide what constitutes a sufficient sample. Data saturation (ie, the point at which no new themes or ideas are identified) is a common method of determining a sufficient sample [82]. In research designs that use free-response qualitative surveys, a median sample size of 75 has previously been identified as required for reaching saturation [82]. Therefore, a minimum sample of 75 is sought.

#### Eligibility

The eligibility was the same as specified for study 1 above.

#### Measures

**Figure 1:**
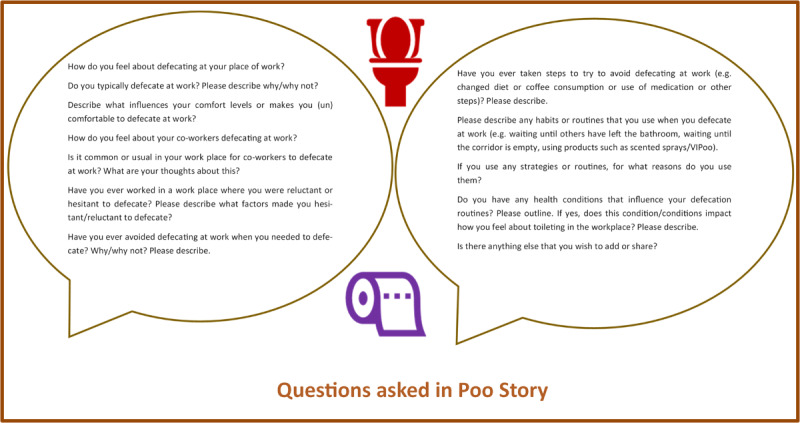
Questions regarding attitudes, patterns, behaviors, and strategies used at a respondent’s place of employment.

Study 2 collates descriptive information pertaining to age and employment status. Respondents are asked a series of 12 questions, some with probes, each with a separate free-text box without a response character limit. Specific questions used in the survey are displayed in Figure 1.

## Results

### Description of Sample 1

Study 1 commenced enrollment in January 2019 and finished enrollment in December 2023. The analyses to answer the study’s hypotheses will commence after the publication of the protocol, expected in the first quarter of 2025. Thematic analyses are expected to occur in the second half of 2025. From 2113 persons who read the study information sheet, 241 were ineligible for the following reasons; withdrawing consent (n=5), not answering any questions (n=131) and not completing the assessment of bowel or psychosocial factors (n=105) leaving an analytical sample of 1872 persons and estimated 88.6% completion rate (Table 1). It should be noted that a further 95 persons did not rate their work or home stool type on the Bristol Stool Form and Frequency chart but had sufficient data for occupational and residential bowel movement frequency.

The sample was comprised predominantly of women (1413/1872, 75.5%), and the average (SD) age was 34.5 (SD 11.3) years. Approximately 29.5% (552/1872) of respondents had a household income above the Australian median income. However, 3.5% (66/1872) of respondents’ work was outside of Australia, and, in total, workers in 27 countries completed the study, most commonly from Australia and New Zealand (1788/1872, 95.5% of the sample).

The occupational characteristics of study 1 indicate that most respondents were engaged in full-time employment (962/1872, 51.4%), with work hours mostly between 31 and 45 hours per week (897/1872, 47.9%; Table 2). Employees had been in their current place of employment for an average 7.7 (SD 8.1) years. The majority of respondents were not engaged in shift work or working during the biological night (1264/1872,67.5%) with the remaining 32.5% (608/1872) engaged in some form of shift work. A total of 19.7% (369/1872) of respondents were classified as working during the biological night.

**Figure 2:**
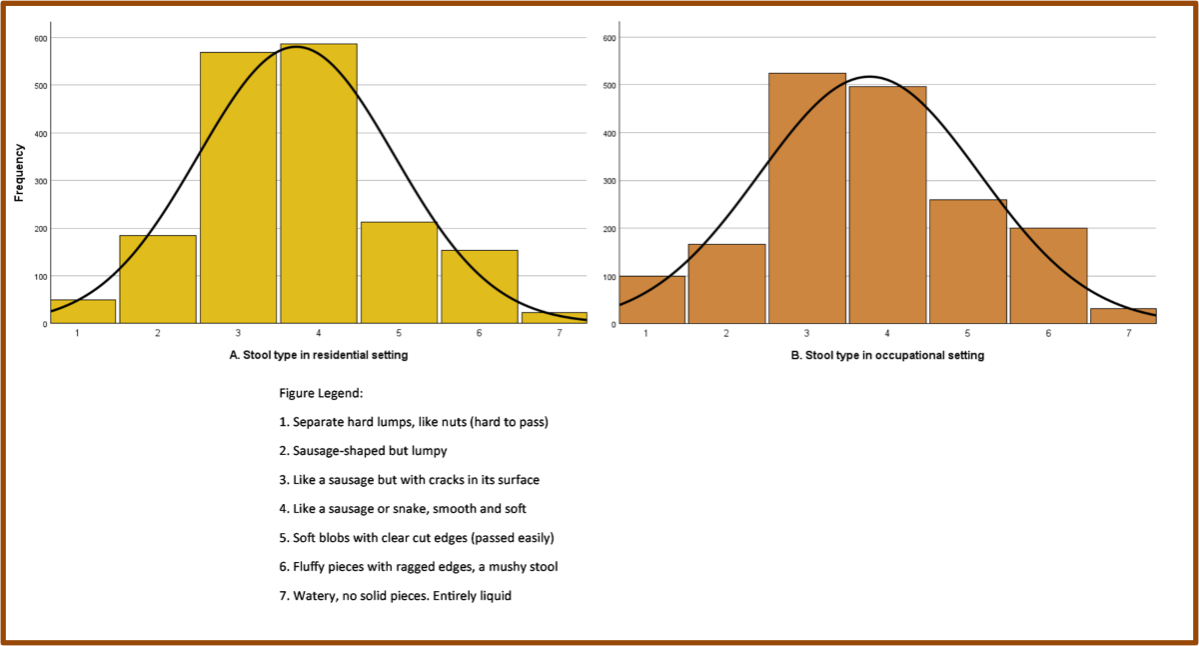
A histogram depicts the frequency of different types of stool selected by participants for the most recent home (panel A) and work (panel B) bowel movement. Participants selected the most appropriate response using a visual representation and description of the Bristol Stool Form Chart.

A graphical display of respondents at home and occupational bowel movements (Figure 2) indicates that type 3 (like a sausage but with cracks in its surface) and type 4 (like a sausage or snake, smooth and soft) bowel movements were the most common, irrespective of location.

**Table 1:** Descriptive information of ITS POO TIME (Insight inTo Stress and POOping on work TIME) study 1.

Descriptive information				Completed study (N=1872)^a^				
Age (years)^b^, mean (SD), IQR				34.5 (11.3), 26-42				
**Gender, n (%)**								
		Women	1413 (75.5)					
		Men	449 (24.0)					
		Nonbinary and other gender identities	10 (0.5)					
**Household income (Aus $), n (%)**								
		0-50,000 (US $34,390)			428 (22.9)			
		50,000-100,000 (US $34,390-69,880)			631 (33.7)			
		100,000-150,000 (US $69,880-104,820)			413 (22.1)			
		> 150,000 (US $104,820)			400 (21.4)			
**Region where working, n (%)**								
	Australia and New Zealand			1787 (95.5)				
	Europe			40 (2.1)				
	North America			17 (0.9)				
	South America			12 (0.6)				
	Africa and Middle East			6 (0.3)				

^a^Data presented as n (%) unless otherwise specified.

^b^Data presented as mean (SD) followed by IQR.

**Table 2:** Employment characteristics of ITS POO TIME (Insight inTo Stress and POOping on work TIME) study 1.

Employment characteristics		Completed study (N=1754)^a^
**Employment status, n (%)**		
	Full-time	962 (51.4)
	Part-time	491 (26.2)
	Self-employed	114 (6.1)
	Casual	305 (16.3)
**Work hours per week, n (%)**		
	0-15	243 (13)
	16-30	488 (26.1)
	31-45	897 (47.9)
	46-60	207 (11.1)
	>60	37 (2)
Years in current employment, mean (SD), IQR		7.7 (8.1), IQR 2-11
**Number of employees at workplace, n (%)**		
	Sole (self-employed)	117 (6.3)
	Small ≤10	363 (19.4)
	Medium 11 to 50	555 (29.6)
	Moderate 51 to 100	274 (14.6)
	Large 101 to 1000	347 (18.5)
	Very large ≥ 10,001	216 (11.5)
**Work during the biological night, n (%)**		
	9 PM to 4 AM	199 (10.6)
	4 AM to 7 AM	146 (7.8)
**Commute time to employment (mins), n (%)**		
	≤15	833 (44.5)
	16-30	553 (29.5)
	31-60	374 (20.0)
	>61	112 (6.0)

^a^Data presented as n (%).

### Description of Sample 2

Study 2 commenced enrollment in December 2019 and finished enrollment in September 2020. A total of 346 persons commenced the study. Of those who responded to study 2, 3 did not provide consent and were excluded, 8 persons did not respond to any of the open-ended questions, and 1 person provided part responses to some of the open-ended questions. Due to the qualitative nature of the analysis, any completed data will be retained for thematic analysis, resulting in a final analytical sample of 337 for study 2. The average age was 34 (SD 10.49) years. Of the analytical sample, 49.6% (167/337) worked full time, 34.7% (117/337) were engaged in part-time work, and 10.1% (34/337) undertook shift work, while 0.9% (3/337) worked both full-time and a part-time job, and 4.7% (16/337) did not specify their employment type.

## Discussion

### Principal Results

This will be the first research project known to the authors with the specific aim of assessing bowel movement anxieties and DGBI in shift and nonshift workers. The qualitative study aims to identify attitudes to bowel movements in occupational settings as well as patterns, behaviors, and strategies used to mitigate embarrassment, fear, or parcopresis. The research study is pivotal in providing further information regarding the prevalence of parcopresis and its relationship to occupational toileting avoidance as well as gastrointestinal health in a range of employees, including shift and nonshift workers.

### Limitations

ITS POO TIME is limited in scope to persons with employment, meaning that the findings may not generalize readily to persons engaged in nonpaid internships, persons studying, or those with carer responsibilities. A related limitation is that we have restricted the research focus to bowel movements in occupational settings when parcopresis applies more generally to the use of any public restroom. It remains unknown and a limitation of ITS POO TIME whether parcopresistic fears of embarrassment are amplified around known persons such as work colleagues compared with strangers.

In the interests of brevity, the assessment battery is limited to subsets of more exhaustive questionnaires. Indeed, we have adopted brief measures and subscales for key constructs in our study pertaining to social anxiety, contamination fears, and parcopresis. With regards to the latter, although this construct is commonly comorbid with paruresis, our study has not assessed paruresis. More than 50% of persons with this condition report limiting their occupation because of paruresis [83]. This may have a significant impact on the research findings and although there are known gender differences in paruresis [60], it remains unclear whether gender differences hold in parcopresis. The study 1 sample is overly representative of female gender respondents which may lead to biases in planned comparisons across genders, while study 2 did not collate information on gender. A possible explanation for the overrepresentation of female gender respondents could relate to oversampling from specific occupations characterized by shift work (eg, nurses) and toileting restrictions (eg, school teachers). Another limitation is that there are potential selection biases and response biases and we were unable to reach the prespecified power calculation. This study did not reach the target sample as stipulated in the power calculation due to larger survey response attrition than anticipated combined with an institutional change in ethics and recruitment procedures, resulting in a need to close the study after 4 years. Consequently, the results will result in less than 90% power to detect a difference in DGBI between shift work groups.

### Conclusions

There is a known paucity of empirical data concerning parcopresis [60]. This project will offer novel insights into occupational, behavioral, and psychosocial factors associated with gastrointestinal health and identify if there are differences between residential and occupational toileting frequency and voiding behaviors. The results of the studies projected in this research protocol will have direct implications for industry, employers, and policy makers concerning functional gastrointestinal diseases, stress, and worker health.

## Data Availability

Data generated or analyzed during this study are subject to an embargo of 12 months from the publication date of the article. Once the embargo expires the data will be available upon reasonable request from the corresponding author on reasonable request, for noncommercial purposes, and without breaching participant confidentiality.

## Appendix

Multimedia Appendix 1 Checklist for Reporting Results of Internet E-Surveys (CHERRIES).

## Abbreviations

CHERRIES: Checklist for Reporting Results of Internet E-Surveys
DGBI: disorders of gut-brain interactions
FFQ: Food Frequency Questionnaire
ITS POO TIME: Insight inTo Stress and POOping on work TIME
K10: Kessler Psychological Distress Scale
Mini-SPIN: Mini-Social Phobia Inventory
R4DQ: Rome IV Diagnostic Questionnaire
SCI: Sleep Condition Indicator
UNE: University of New England
