# Supplementary material for: Insight inTo Stress and POOping on Work TIME (ITS POO TIME): Protocol for a Web-Based, Cross-Sectional Study
Source: JMIR Res Protoc. 2025 Jun 5;14:e58655. doi: 10.2196/58655 (PMC12179570; doi:10.2196/58655)
Supplement: Multimedia Appendix 1 [file resprot_v14i1e58655_app1.docx]

**Supplement 1: Checklist for Reporting Results of Internet E-Surveys (CHERRIES) (1)**

| ***Checklist Item*** | ***Explanation*** | ***Page Number*** |
| --- | --- | --- |
| Describe survey design | The study uses a convenience sample. | 7 |
| IRB approval | Ethics approval was granted at two tertiary institutions. | 7 |
| Informed consent | Potential participants were informed of the purpose of the survey, anonymity, confidentiality, and voluntary principles before responding. | S1 |
| Data protection | No personal identifying information was collected. Only PI Tully had access to the surveymonkey data to protect unauthorized access. A de-identified and cleaned dataset is hosted on institutional servers in accordance with a data management plan. | S1 |
| Development and testing | The questionnaire was tested by 10 university academics and students to identify issues with formatting, page logic, and scoring algorithms. | S1 |
| Open survey versus closed survey | This is an open survey. | S1 |
| Contact mode | Initial contact was made by filling in the questionnaire via the recruitment strategy. Potential responders were not contacted directly or emailed. | S1 |
| Advertising the survey | The survey information was listed on the University of New England Faculty of Health research participant pool website and electronic flyers in the Faculty of Health at The University of Adelaide. Additional recruitment was supplemented by promotional interviews with radio (ABC Adelaide, ABC New England) and free to air television stations (PRIME 7 News New England), and organisations representing occupations with shift work (Nursing Times) or known toileting restrictions (NSW Teachers Federation). | 7, S1 |
| Web/E-mail | This is a website survey hosted on an electronic platform surveymonkey. | S1 |
| Context | This is a website survey hosted on an electronic platform surveymonkey. | S1 |
| Mandatory/voluntary | Participation was voluntary. | S1 |
| Incentives | There were no incentives offered. |  |
| Time/Date | Study 1 commenced enrolment in January 2019 and finished enrolment in December 2023. Study 2 commenced enrolment in December 2019 and finished enrolment in September 2020. | 14, 17 |
| Randomization of items or questionnaires | The survey was presented in blocks starting with demographic and occupational information. Thereafter, pages were randomized. | S1 |
| Adaptive questioning | The Rome Foundation screener for Disorders of Gut Brain Interaction uses adaptive questioning and question logic built in to the survey to reduce the number and complexity of the questions. | 10 |
| Number of Items | There were approximately 11 questions per page. | S1 |
| Number of screens (pages) | There was 9 pages in total including information sheet and consent. | 19 |
| Completeness check | Post survey results have a completeness estimate. No question was mandatory and respondents could opt not to answer an item if desired. | S1 |
| Review step | Respondents were not able to review their responses after submitting responses at the end of the survey. Prior to submission, respondents could navigate between pages and alter their responses at any time. | S1 |
| Unique site visitor | We did not collect data on site visits. | S1 |
| View rate (Ratio of unique survey visitors/unique site visitors) | We did not collect data on site visits. | S1 |
| Participation rate (Ratio of unique visitors who agreed to participate/unique first survey page visitors) | The participation rate is calculated as; 2108 persons viewed the first page of the survey / from 2113 who completed the consent sheet = 99%. | S1 |
| Completion rate (Ratio of users who finished the survey/users who agreed to participate) | The attrition rate is calculated as; 1872 / 2113 = 88.6% | 15, S1 |
| Cookies used | Cookies were not utilized. | S1 |
| IP check | An IP address was recorded to identify potential duplicate entries from the same user within a 24 hour period. Instances of duplicate entry the most recent entry would be considered, however, there were no duplicate entries identified. | S1 |
| Log file analysis | None. | S1 |
| Registration | Not applicable, open survey. | S1 |
| Handling of incomplete questionnaires | We excluded persons who did not complete questions pertaining to parcopresis a key outcome. Response to 50% or more of the survey was considered a partial completer and retained for analysis. | S1 |
| Questionnaires submitted with an atypical timestamp | Timestamp responses less than 5 minutes were ineligible, however, there were no completer responses identified. | S1 |
| Statistical correction | The study recruited 75% persons identifying as female gender. We will de-aggregate data by gender rather than weight the analyses. | S1 |

**Reference**

1. Eysenbach G. Improving the Quality of Web Surveys: The Checklist for Reporting Results of Internet E-Surveys (CHERRIES). J Med Internet Res. 2004;6(3):e34.
